# Bioactive Peptides Derived from Tuna: Screening, Extraction, Bioactivity, and Mechanism of Action

**DOI:** 10.3390/md23070293

**Published:** 2025-07-21

**Authors:** Jing-an Cheng, Di Wang, Gang Yu, Shengjun Chen, Zhenhua Ma, Ya Wei, Xue Zhao, Chunsheng Li, Yueqi Wang, Yi Zhang, Rong Cao, Yongqiang Zhao

**Affiliations:** 1 Key Laboratory of Aquatic Product Processing, Ministry of Agriculture and Rural Affairs, South China Sea Fisheries Research Institute, Chinese Academy of Fishery Sciences, Guangzhou 510300, China; m230351157@st.shou.edu.cn (J.-a.C.);; 2 College of Food Science & Technology, Shanghai Ocean University, Shanghai 201306, China; 3 Key Laboratory of Efficient Utilization and Processing of Marine Fishery Resources of Hainan Province, Sanya Tropical Fisheries Research Institute, Sanya 572426, China; 4 Department of Food Science, The Pennsylvania State University, University Park, PA 16802, USA; 5 Yellow Sea Fisheries Research Institute, Chinese Academy of Fishery Sciences, Qingdao 266071, China

**Keywords:** tuna, bioactive peptides, antioxidant, antihypertensive, human health

## Abstract

Peptides play a crucial role in the development of pharmaceuticals and functional foods. Multiple studies have shown that natural bioactive peptides possess antioxidant, antihypertensive, anti-tumor, and anti-inflammatory activities. Marine bioactive peptides, especially those sourced from fish, constitute a substantial reservoir of these molecules. Although considerable research has been undertaken on fish-derived peptides, studies specifically concerning those from tuna are limited. Tuna, a marine fish of high nutritional value, generates substantial by-product waste during fishing and processing. Therefore, it is essential to conduct an evaluation of the advancements in study on tuna-derived active peptides and to offer a perspective on the direction of future investigations. This review integrates prospective bioactive peptides derived from tuna and reports contemporary strategies for their investigation, including extraction, purification, screening, identification, and activity evaluation procedures, including Yeast Surface Display (YSD) and molecular docking. This review seeks to promote the continued investigation and application of bioactive peptides derived from tuna.

## 1. Introduction

Tuna, a highly migratory species, populates the Pacific, Atlantic, and Indian Oceans and plays a significant role in global fisheries. Of the approximately 15 tuna species, bluefin, yellowfin, and bigeye are the most commercially valuable [1]. Tuna is rich in Docosahexaenoic Acid (DHA) and Eicosapentaenoic Acid (EPA), making it a nutritious food, commonly available as canned fish, raw fish, and fish oil [2,3]. Recent data from the Food and Agriculture Organization of the United Nations (FAO) and regional fisheries management organizations indicate that the global annual tuna catch has stabilized between 5 and 6 million tons. Tuna processing produces many by-products, such as fish blood, bones, fish skin, etc., resulting in waste of resources and environmental pollution. Utilizing these by-products could offer health benefits [4].

Most bioactive peptides consist of 2 to 20 amino acids [5]. They exhibit diverse biological activities influenced by their sequence, composition, and structure [6]. Some of these polypeptides can be synthesized by the organism itself, while others need to be ingested through external routes, they are absorbed more efficiently than free amino acids and enhance the sensory attributes of food. Notably, bioactive peptides do not accumulate in organisms and have minimal side effects. Consequently, they hold significant potential in functions such as antioxidant, antibacterial, antihypertensive, and analgesic activities [7]. Recent studies highlight marine bioactive peptides for their promising anti-tumor [8], anti-infection [9], hypoglycemic [10], and hypotensive properties.

In recent years, increasing focus has been directed towards extracting bioactive peptides from food, particularly processed by-products, as potential supplements or replacements for drugs in disease treatment. Extensive research into the mechanisms of bioactive peptides has underpinned the development of highly effective, side-effect-free drugs. Significant advances have been made in studying tuna protein. For instance, Sang-Hong Lee and colleagues identified peptides with high angiotensin-converting enzyme (ACE) inhibitory activity through tuna protein hydrolysis [11], which effectively lowers blood pressure. Similarly, Kou-Chiang Hsu and co-researchers extracted peptides with high antioxidant activity from tuna dark muscle by-products [12]. This paper aims to introduce bioactive peptides related to tuna, detailing their mechanisms of action, extraction, and screening methods.

## 2. Bioactive Peptide from Tuna

Tuna bioactive peptides are small molecules with distinct biological activities, derived from tuna proteins through enzymatic or chemical hydrolysis, and microbial fermentation. Typically sourced from tuna muscle, skin, and internal organs. Tuna itself has a high amino acid content, and the proportion of hydrophobic amino acids is significantly higher than that of terrestrial peptides. Studies have shown that the distribution of hydrophobic amino acids at the N-terminal and C-terminal can significantly enhance the targeted binding ability and stability of peptides [13].These peptides offer promising applications in food science, nutrition, and medicine. The subsequent sections will explore the specific functions and mechanisms of these tuna-derived bioactive peptides.

### 2.1. Antioxidant Peptides

#### 2.1.1. Mechanism of Action of Antioxidant Peptides

Antioxidant peptides, due to their lower molecular mass, are more effective than their parent proteins in inhibiting free radical-mediated peroxidation. This is because longer peptide chains may disrupt the protein structure and hinder antioxidant active amino acid residues. The antioxidant capacity of natural compounds is typically assessed via two mechanisms: free radical scavenging and metal ion chelation [14]. In the human body, free radical production and clearance are balanced. However, excessive free radicals can damage proteins, DNA, and lipids, leading to various diseases [15]. Antioxidant peptides neutralize free radicals by donating hydrogen atoms or electrons, stabilizing these reactive species. They also inhibit oxidation-promoting enzymes. Elevated reactive oxygen species (ROS) levels increase stress hormones, triggering enzymes, cortisol, and catecholamine release. Over time, oxidative processes cause nutrient degradation, unpleasant odors, and reduced product shelf life [16]. Consuming adequate antioxidants can prevent or mitigate ROS-induced oxidative damage [17]. Butylated hydroxytoluene (BHT), butylated hydroxyanisole (BHA), and tert-butylhydroquinone (TBHQ) are widely used synthetic antioxidants, yet they pose safety concerns [18]. In contrast, endogenous biological antioxidants effectively mitigate free radicals and reactive oxygen species (ROS) generated by metabolic processes, thereby protecting macromolecules like DNA, proteins, and lipids from damage [19]. Given their superior safety profile, natural antioxidants have emerged as a significant focus of research.

**Figure 1:**
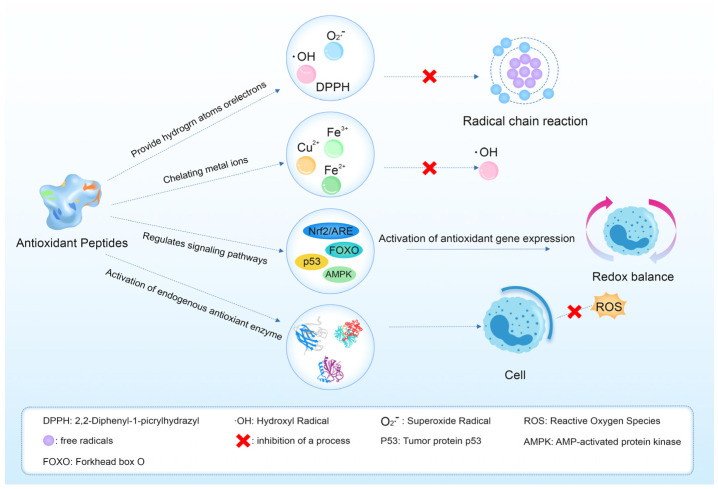
Mechanism of antioxidant peptides.

Antioxidant peptides neutralize free radicals, inhibit enzyme activity and oxidative reactions, and protect cell membranes, thereby preserving cellular function, reducing inflammation, and potentially preventing diseases linked to oxidative stress. Deep-sea fish, rich in omega-3 fatty acids like EPA and DHA [20], influence oxidation rates and signal transduction, decrease cholesterol accumulation in cell membranes, and mitigate oxidative stress damage [21]. The mechanism of action of antioxidant peptides is shown in Figure 1.

The following introduces some research progress of tuna source antioxidant peptides.

#### 2.1.2. Antioxidant Peptides in Tuna

Currently, antioxidant peptides have been extracted from various fish species. For instance, Wiriyaphan C et al. [22] isolated antioxidant peptides from threadfin bream. Similarly, peptides with antioxidant properties have been derived from silver carp [23], rainbow trout [24], and other commonly studied fish. Comparable results have also been observed in tuna research.

Studies indicate that antioxidant peptides from tuna head enhance Glutathione Peroxidase (GSH-Px) activity and lower Malondialdehyde (MDA) levels in serum and liver. Glutathione, a critical marker of redox status in various diseases and aging, functions as an antioxidant, protects against cellular damage, and regulates metabolism [25]. It interacts with numerous enzymes and proteins. Glutathione peroxidase, a key enzyme in antioxidant defense, removes toxic peroxides, mitigating oxidative stress-induced cellular damage. Its activity reflects a cell’s oxidative stress sensitivity and resistance [26]. MDA, a lipid peroxidation byproduct from free radical attack on unsaturated fats, serves as a common oxidative stress marker. MDA levels indicate lipid peroxidation extent, thus indirectly reflecting antioxidant enzyme activity and efficacy in the body [27]. Studies have demonstrated that collagen peptides from bigeye tuna skin and bone can effectively eliminate excess reactive oxygen species and decelerate skin aging by activating the TGF-β1 signaling pathway while inhibiting the MAPK pathway [28]. Wardani et al. [29] corroborated these findings, also identifying antioxidant peptides in yellowfin tuna skin collagen hydrolysate. These peptides competitively inhibit myeloperoxidase (MPO) by blocking its active site, thereby exhibiting antioxidant properties. Cai et al. identified similar peptides from tuna protein that interact with MPO to cover its active site and inhibit its catalytic activity [30]. Additionally, researchers processed tuna fish bones to produce gelatin, which was further purified to isolate various antioxidant peptides [31]. Saidi et al. isolated four novel antioxidant peptides from tuna by-products using gel chromatography and reversed-phase HPLC. These peptides, exhibiting the highest antioxidant activity, have the sequences YENGGG, EGYPWN, YIVYPG, and WGDAGGGYY [32]. The molecular weights of the peptides were 538.46, 764.75, 710.78, and 887.85 Da, respectively. Je et al. [33] treated tuna cytoskeleton with pepsin, yielding an antioxidant peptide of 1519 Da that effectively scavenged free radicals and inhibited lipid peroxidation. Acid hydrolysis was employed to extract gelatin from tuna skin, and the resulting hydrolysate, treated with neutral protease, showed strong DPPH scavenging activity. Peptides smaller than 3.5 kDa, obtained through filtration, protected human skin fibroblasts from Ultraviolet A (UVA) damage, with 19 of these peptides demonstrating antioxidant activity [34]. J Wang et al. [35] hydrolyzed bonito eggs using flavor protease, producing a hydrolysate with significant DPPH scavenging ability. Twelve antioxidant peptides, each under 1 kDa, were subsequently isolated via ultrafiltration and chromatography, with potential applications in drug and functional product development.

Bioactive peptides from living organisms can be harnessed for use in food and medicine. Unlike conventional drugs, these natural peptides typically have fewer side effects and are safe for human health. The beauty industry has already advanced in applying antioxidant peptides, indicating substantial potential for utilizing antioxidant peptides from tuna by-products. The specific information of antioxidant peptides is shown in Table 1.

### 2.2. Hypotensive Active Peptides

#### 2.2.1. Mechanism of Action of Hypotensive Active Peptides

**Figure 2:**
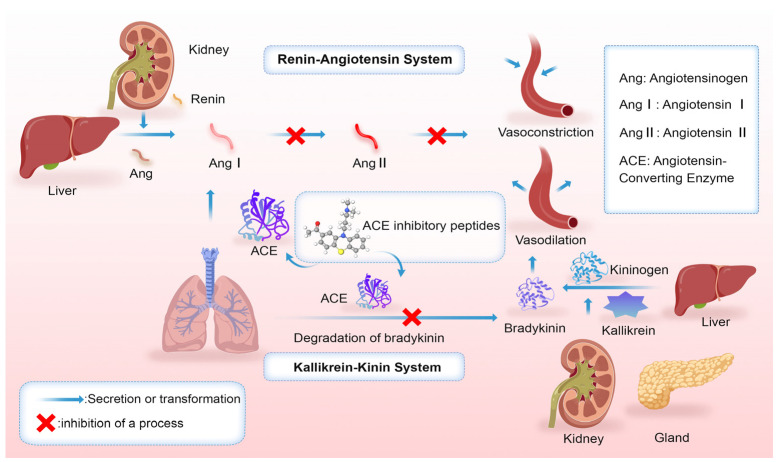
Mechanism of ACE inhibitor peptides (RAS and KKS).

Hypertension, a chronic and multifactorial disease, is a leading cause of premature mortality, responsible for over 9 million deaths annually and affecting around 1 billion individuals globally [36]. In 2000, approximately 972 million people were affected, with projections suggesting this will increase to 1.56 billion by 2025 [37]. Maintaining normal blood pressure can delay or prevent complications, regulated through mechanisms such as vasodilation, ACE inhibition, and increased urine output. Common antihypertensive treatments include agents like captopril, enalapril, and lisinopril [38]. The renin–angiotensin system (RAS) and the kallikrein–kinin system (KKS) are crucial for regulating blood pressure, hemostasis, inflammation, and renal function [39]. ACE converts angiotensin I to angiotensin II by removing the C-terminal dipeptide His-Leu, with angiotensin II acting as a potent vasoconstrictor that elevates arterial pressure [40]. In the KKS, kallikrein processes kininogens to form bradykinin, promoting vasodilation and reducing blood pressure. However, ACE degrades bradykinin inhibiting its vasodilatory effect and contributing to increased blood pressure. Elevated ACE activity disrupts the equilibrium between pressor and hypotensive mechanisms, leading to excessive angiotensin II production and diminished bradykinin and prostaglandin synthesis, which dilate blood vessels. The regulation mechanisms of ACE by the KKS and the RAS are shown in Figure 2.

Antihypertensive peptides exhibit diverse mechanisms of action. Atrial and brain natriuretic peptides enhance diuresis and sodium excretion, thereby decreasing fluid volume and blood pressure. Crucially, blood pressure control often involves reducing ACE activity, as ACE-inhibitory peptides block angiotensin II synthesis, thus lowering blood pressure. Nonetheless, industrially produced ACE inhibitors face challenges, and conventional antihypertensive drugs often have side effects. Researchers are striving to develop healthier, more accessible alternatives [41].

#### 2.2.2. Hypotensive Active Peptides in Tuna

Researchers have isolated ACE-inhibiting peptides from various aquatic species, such as grass carp [42], cuttlefish [43], and squid [44]. Seki et al. hydrolyzed 12 food proteins and discovered that ACE inhibitory peptides from fish, shrimp, crab, and other aquatic animal proteins exhibited superior antihypertensive activity compared to other food proteins [45]. Using computational tools, including molecular docking and molecular dynamics simulations, along with in vitro experiments, Wang et al. identified two stable, novel ACE inhibitory peptides from tuna [46]. Additionally, Wang extracted another peptide, LTGCP, from tuna muscle via a two-step hydrolysis method. However, unlike the previous peptides, LTGCP’s effects have not been evaluated in vivo, limiting its persuasive power [47]. R Intarasirisawat et al. extracted and isolated antioxidant and ACE inhibitory peptides from skipjack roe, identifying the peptide MLVFAV as having the highest ACE inhibitory activity [48]. Zu et al. developed an affinity medium with a potential application for the efficient screening of ACE inhibitory peptides and successfully identified active peptides with hypotensive effects from tuna proteins using this medium [49]. Qian et al. [50] isolated an angiotensin I-converting enzyme (ACE) inhibitory peptide from the hydrolysate of bigeye tuna dark muscle, demonstrating notable antihypertensive effects. Qiao et al. identified 14 ACE inhibitory peptides in skipjack tuna by-product hydrolysate, including TE, AG, MWN, MEKS, VK, MQR, MKKS, VKRT, IPK, YNY, LPRS, FEK, IRR, and WERGE. Extracting ACE inhibitory peptides from processing by-products enhances tuna utilization and aids hypertension treatment [51]. Hydrolyzed tuna cooking juice exhibited the strongest ACE inhibitory and calcium-binding activities after 270 and 180 min of hydrolysis, respectively. Peptides with significant ACE inhibitory and calcium-binding activities had molecular weights of 238–829 Da and 1355–188 Da [52]. Ten novel ACE-inhibiting peptides were identified in skipjack tuna by-product hydrolysate using alkaline protease. Among them, peptides ICY, LSFR, and IYSP showed strong ACE inhibitory effects and significantly protected H_2_O_2_-damaged HUVECs by enhancing antioxidant enzyme levels and reducing reactive oxygen species and malondialdehyde [53]. Studies indicate that ICY, LSFR, and IYSP are effective molecules for treating hypertension and cardiovascular diseases. Similarly, an active peptide from skipjack tuna muscle protein identified by Zheng et al. demonstrated ACE inhibition [54]. They isolated six ACE-inhibitory peptides from muscle protein hydrolysates, with SP and VDRYF showing notable effects. Hypertension, a prevalent chronic condition, often necessitates long-term medication to manage blood pressure. Bioactive peptides with ACE-inhibitory properties present a promising alternative to conventional drugs, potentially mitigating pharmaceutical side effects. Research on ACE-inhibitory peptides and antihypertensive components from tuna are gaining significant attention. The detailed information of ACE inhibitory peptides is shown in Table 2.

### 2.3. Other Bioactive Peptides in Tuna

**Figure 3:**
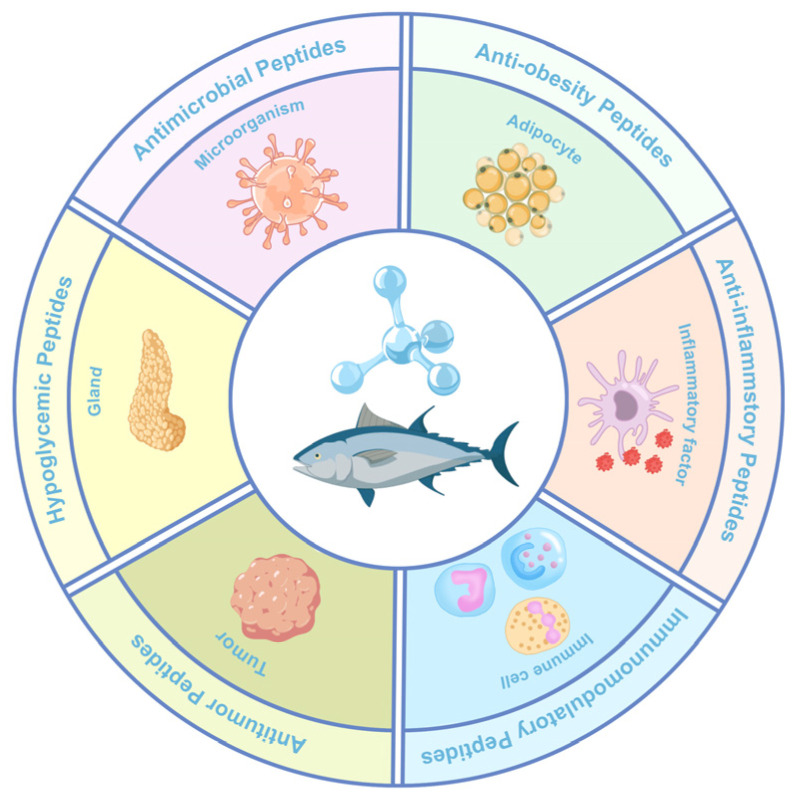
Bioactivity of tuna active peptides.

Beyond antioxidant and antihypertensive peptides, organisms possess a variety of biologically active peptides, including those that lower lipids and glucose, as well as antimicrobial and food supplement peptides. Marine organisms, such as fish, seaweed, shellfish, microalgae, crustaceans, cephalopods, and mollusks, are abundant sources of proteins and bioactive peptides [55]. In addition to ACE inhibitory activity and antioxidant activity, tuna active peptides have a variety of biological activities such as antimicrobial, anticancer, and anti-obesity, as shown in Figure 3.

Tansukkasem et al. [56] isolated heme iron polypeptide (HIP) from tuna blood. High-performance liquid chromatography (HPLC) analysis revealed that this low-hydrolytic tuna HIP has a molecular weight ranging from 1 kDa to 5 kDa, suggesting its potential as a food iron supplement. Antimicrobial peptides (AMPs) are promising antibiotic alternatives due to their broad-spectrum antibacterial activity, thermal stability, and low risk of resistance [57]. Research indicates that AMPs exert antibacterial effects by interacting with and disrupting bacterial cell membranes [58]. Fish are significant sources of AMPs, expressing various types such as hepcidins, cathelicidins, and histone-derived peptides. These AMPs not only eliminate pathogens but also modulate immune responses. Consequently, extracting AMPs from fish is crucial in developing antimicrobial therapeutics [59]. Seo J K et al. [60] initially isolated an antimicrobial peptide (AMP) from the skin of the yellowfin tuna, naming it YFGAP. YFGAP exhibited resistance to both Gram-positive and Gram-negative bacteria, including *A. hydrophila*, *S. iniae*, and *V. parahemolyticus*, indicating that tuna may also be a source of antimicrobial peptides. Zhao et al. found that tuna hydrolysate might alleviate the immunosuppression and intestinal mucosal damage caused by 5-FU and also had the potential to fight tumors [61]. In tuna black muscle by-products hydrolyzed with papain and protease XXIII, two peptides were identified: LPHVLTPEAGAT (1206 Da) and PTAEGGVYMVT (1124 Da). These peptides, with IC50 values of 8.1 μM and 8.8 μM, respectively, inhibited the proliferation of human breast cancer cells, suggesting that tuna by-products could serve as potential sources of anti-proliferative peptides [62]. The alkaline protease hydrolyzed bonito dark muscle, producing a hydrolysate named KPHs-AL, which modulates intracellular lipid accumulation by interacting with phospholipids. This interaction significantly reduces triglyceride (TG) levels, thereby decreasing lipid accumulation and the risk of obesity [63]. The antibacterial peptide named SHβAP, purified from the liver extract of skipjack tuna, has certain inhibitory effects on a variety of bacteria [64]. Yu-rou et al. [65] and co-researchers identified trypsin as the optimal hydrolase for preparing active peptides with uric acid-lowering properties. Wu et al. [66] and team confirmed the anti-inflammatory and cartilage-protective effects of Skipjack tuna elastin peptides (STEP), suggesting their potential in treating osteoarthritis (OA). Research on xanthine oxidase inhibitory peptides derived from tuna protein is ongoing [67].Tyrosinase (TYR) inhibitors in collagen hydrolysate from marine by-products effectively inhibit melanin production, offering potential applications in the pharmaceutical and cosmetic industries [68]. Studies have shown that the intake of tuna extract containing imidazole compounds can reduce the level of uric acid in serum [69]. Some tuna active peptides also have the effect of lowering uric acid. The research results of Wu et al. show that the elastin peptides of bonito tuna can change the composition and distribution of the microbiota in the intestinal tract of mice, keeping the intestinal tract of mice in a relatively healthy state [70]. Researchers have found that the hydrolysate of tuna meat can alleviate the symptoms of hyperuricemia in mice, and at the same time proposed that peptides containing Phenylalanine have the potential to combat hyperuricemia [71]. The peptides prepared by Hao et al. from yellowfin tuna demonstrated good anti-hyperuricemia activity by influencing purine metabolism. Meanwhile, the peptide also had certain protective effects on the liver and kidneys of mice [72]. Bu et al. used a network platform to screen out peptides with xanthine oxidase inhibitory activity from bluefin tuna myosin. The mechanism of action is to prevent xanthine from entering the active site of XOD [73]. The buffer solution also has a certain influence on the activity of tuna xanthine oxidase inhibitory peptide. Studies have shown that the tuna active substance prepared with HEPES as the buffer has a better effect [74]. The table below lists additional bioactive peptides extracted from tuna not previously mentioned.

The overuse of antibiotics has led to a growing threat of drug resistance, posing a significant challenge to the sustainable development of the food industry. Marine bioactive peptides represent a unique source of biologically active compounds that play diverse and essential roles in various biological processes [75]. Marine bioactive peptides offer numerous advantages, including their natural origin, diversity, biological activity, high efficacy, low toxicity, and sustainability. These characteristics make them highly promising for broad applications in the fields of medicine, healthcare, and cosmetics. However, further research and development is needed to fully tap its potential and promote its sustainable use. Table 3 summarizes the relevant information of active peptides.

## 3. Advances in Screening and Extraction Methods for Bioactive Peptides

Various methods are available for the extraction and screening of bioactive peptides, and the selection of appropriate techniques should be based on specific research objectives and experimental conditions.

### 3.1. Extraction of Bioactive Peptides and Purification Methods

Marine bioactive peptides can be synthesized via organic methods, microwave-assisted extraction, chemical, or enzymatic hydrolysis. Enzymatic hydrolysis is favored for its specificity, minimal solvent residue, and mild operational conditions [86]. Protein hydrolysis can be achieved chemically or biochemically. However, chemical hydrolysis can diminish peptide bioactivity, particularly for peptides with unique structures, due to potential amino acid deletions during alkaline treatment [87]. Consequently, despite its simplicity and cost-effectiveness, chemical hydrolysis is seldom used industrially. Enzymatic hydrolysis is now the predominant method for preparing bioactive peptides, utilizing specific enzymes to cleave proteins or peptide chains. Common enzymes include pepsin [88], trypsin [89], chymotrypsin A, collagenase, and degrading enzymes, each with unique hydrolytic properties. By selecting suitable enzymes and optimizing conditions, peptide preparation is enhanced. The hydrolysis process often requires adjustments based on specific needs and experimental parameters. Peptides with different activities have different characteristic amino acids, for example, anti-inflammatory peptides preferably have a basic amino acid at the c-terminus [90], and ACE inhibitory peptides will have increased activity due to the presence of hydrophobic amino acids at the c-terminus [91], so it is necessary to select an enzyme that has certain specific cleavage sites during enzymatic hydrolysis, to increase the likelihood of obtaining peptide segments with certain specific activities. Beyond chemical and enzymatic hydrolysis, bioactive peptides can also be extracted through microbial fermentation and genetic engineering. Microbial fermentation harnesses microorganisms’ metabolic activities to decompose proteins into smaller peptides. Under specific culture conditions, enzyme systems modify and reconstitute peptide groups, generating bioactive peptides. Microorganisms secrete proteases during growth, catalyzing protein hydrolysis and producing bioactive peptides [92]. Rivero-Pino et al. demonstrated that plant-derived peptides from enzymatic hydrolysis and fermentation enhance food systems by boosting nutritional value [93]. The fermentation process involves selecting microbial strains, preparing the medium, conducting fermentation, extracting the broth, purifying peptides, and evaluating bioactivity. Conversely, genetic engineering utilizes genetic technology to produce bioactive peptides. This involves selecting and cloning the target gene, choosing an appropriate host for expression, extracting and purifying the protein, cleaving the target peptide, and assessing its biological activity. The purification of bioactive peptides employs techniques such as chromatography, filtration, electrophoresis, and extraction, often in combination, to achieve effective separation and purification. Membrane-based technologies like microfiltration (MF) and ultrafiltration (UF) separate peptides by molecular weight, addressing diverse purification needs [94]. High-performance liquid chromatography (HPLC) is a widely used multifunctional separation method, with reversed-phase HPLC particularly favored for its operational simplicity, versatility, and ability to process compounds with varying polarity and molecular weight [95]. Currently, the integration of high-efficiency liquid chromatography with high-resolution mass spectrometry is a primary approach for peptide separation and identification. Advances in multi-stage tandem mass spectrometry resolution and data acquisition speed, coupled with ultra-high performance liquid chromatography, enable high-throughput analysis and identification of peptide sequences.

### 3.2. Screening, and Activity Evaluation of Bioactive Peptides

Traditional screening methods for these peptides, such as biological activity assays, cytotoxicity tests, receptor binding assays, random peptide library screenings, and experimental designs, have limitations. These include being time- and labor-intensive, overlooking cytotoxicity, and being constrained by receptor specificity and experimental conditions. Advances in science and technology have introduced high-throughput devices that offer enhanced accuracy, selectivity, and detectability, facilitating the study of bioactive molecules like fatty acids, enzymes, peptides, and amino acids [96].

High-throughput screening of bioactive peptides offers a swift and effective approach to identifying potential candidates. Techniques such as peptide microarray technology, cell-based screening, yeast surface display, and combinatorial library screening are commonly employed. Peptide microarrays utilize solid-phase synthesis to enable the detection of thousands to millions of peptides simultaneously. In cell-based screening, peptides are co-cultured with cells to evaluate their impact on cellular activity.

**Figure 4:**
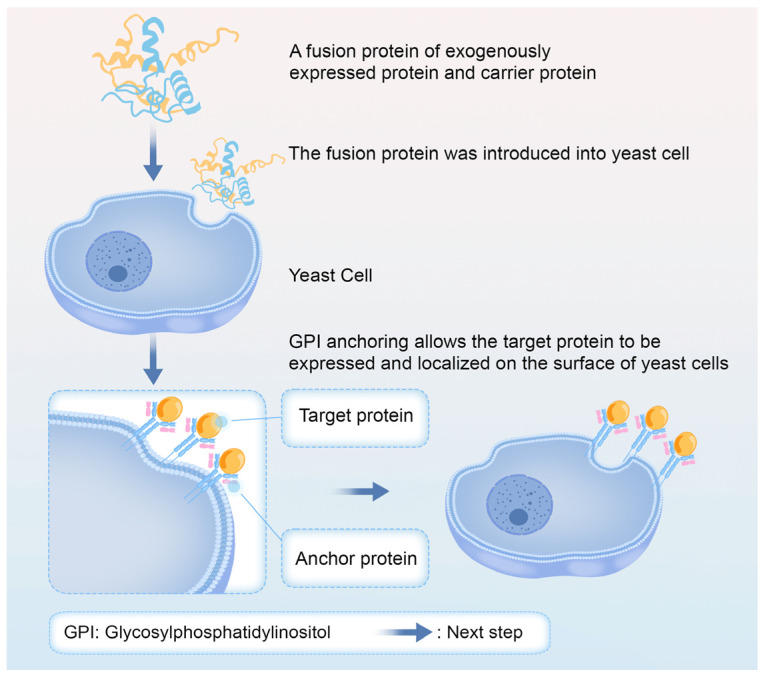
Yeast surface display technique.

Yeast surface display (YSD) facilitates heterologous protein expression on the yeast cell surface by fusing an exogenous protein gene with a vector sequence, which is introduced into yeast cells. Upon expression, a signal peptide directs the fusion protein to the extracellular space, where it is anchored to the yeast cell wall, immobilizing the protein on the surface. This technique not only displays proteins on the cell surface but also allows for their easy fixation and recycling [97]. Figure 4 shows the basic process of YDS technology.

**Figure 5:**
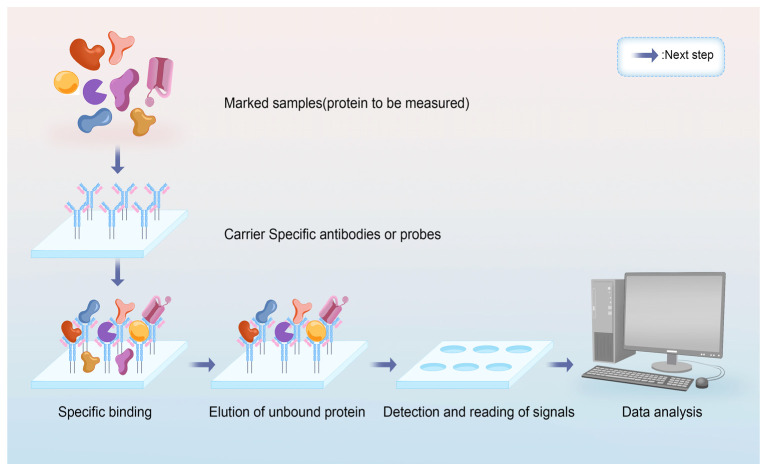
Principle of protein microarray technology.

Protein microarrays serve as high-throughput platforms for the simultaneous analysis of thousands of purified proteins, playing a crucial role in proteomics. They are primarily employed for examining molecular interactions, analyzing post-translational modifications, identifying novel drug targets, and detecting pathogen–host interactions [98]. The preparation of protein chips involves specialized treatment of the solid-phase support to effectively immobilize proteins such as enzymes, antigens, antibodies, receptors, ligands, and cytokines, ensuring their stability and activity on the chip. These molecules, with their diverse properties, facilitate the capture and detection of specific binding proteins. Detection methods in protein microarrays are categorized into labeled and label-free techniques. The specific workflow is shown in Figure 5.

The biological activities of active peptides, their mechanisms, and interactions with enzyme complexes are often assessed through specific in vitro assays and molecular docking studies, enhancing our understanding of peptide functions and roles within organisms [99]. Depending on the peptide and its activity, various methods can be employed for activity determination, with several common techniques outlined below.

Biochemical assays encompass enzymatic and antimicrobial activity assessments, among others. Enzymatic activity in peptides is evaluated by measuring substrate conversion rates or product release during enzyme-catalyzed reactions. Antimicrobial activity is gauged using the Minimum Inhibitory Concentration (MIC), determining the lowest peptide concentration needed to inhibit bacterial growth, thus evaluating antimicrobial potential. Antioxidant peptides are primarily assessed using methods like the DPPH [100] radical scavenging assay, ABTS [101] methodology, and assays for Fe^3+^ and Cu^2+^ reduction. These techniques measure the capacity of antioxidant peptides to scavenge and reduce free radicals through various mechanisms, effectively assessing antioxidant activity.

Receptor binding assays evaluated peptide activity at specific receptors using methods like radiolabeling, fluorescence labeling, and surface plasmon resonance. These techniques determine critical parameters, including binding affinity and rate constants, offering insights into the peptide’s biological activity.

Cytotoxicity and cellular antioxidant activity (CAA) are pivotal in assessing the safety and efficacy of bioactive peptides. The CAA assay offers a significant advantage over in vitro techniques by quantifying antioxidant interactions under physiological conditions, resulting in a more biologically relevant assessment of their vivo efficacy. Cellular models simulate the physiological environment to assess the protective effects of antioxidant peptides against oxidative damage, offering a precise measure of their efficacy [102]. The CAA method, by comparing cellular antioxidant capacity under varying conditions, aligns closely with animal experiment outcomes, making it a robust tool for evaluating antioxidant peptide activity.

**Figure 6:**
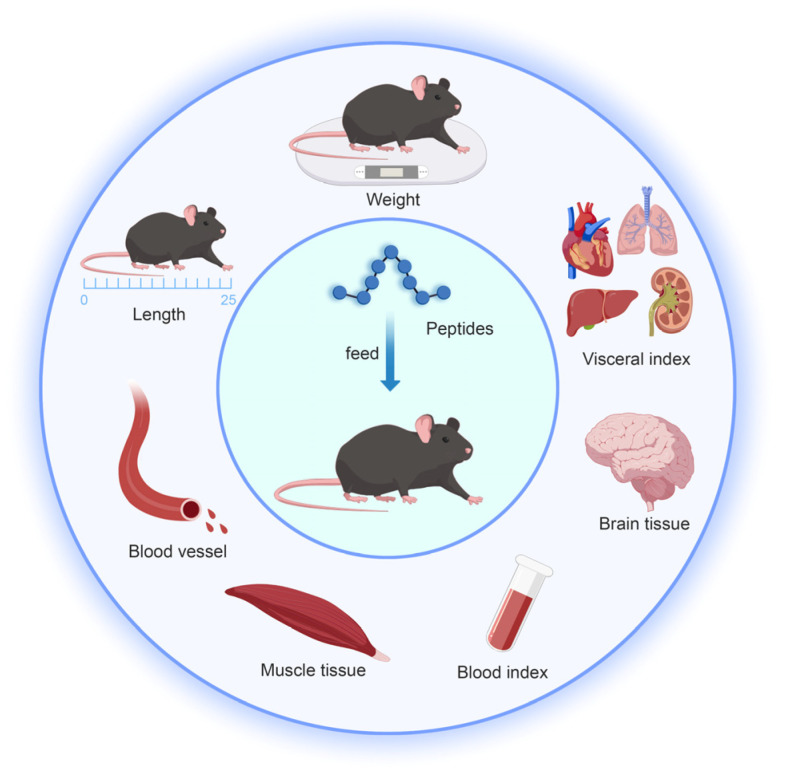
Basic indexes of peptide feeding mice.

The bioactivity was assessed in vivo using animal models, including mice, rats, and zebrafish, to evaluate the biological effects, pharmacokinetic parameters, and safety of peptides. These experiments yield crucial insights into the peptides’ properties and potential applications. Figure 6 illustrates the physiological indicators measured in mice after peptide administration.

Molecular docking is increasingly utilized to simulate molecular interactions, predicting ligand binding modes and affinities with target proteins. It serves as a filter to identify promising research subjects, thereby reducing drug development time and cost. Additionally, it can detect potential drug side effects or molecular toxicity [103]. For instance, docking analysis has elucidated how an antioxidant peptide disrupts the Keap-Nrf2 interaction, promoting Nrf2 accumulation and revealing its antioxidant mechanism [104]. LU et al. utilized molecular docking technology to replace the less active amino acids in the peptide segments with highly active amino acids. The results show that the optimized peptide segment has a better effect [105]. Furthermore, docking studies have shown that FNSL exhibits significant α-glucosidase inhibitory activity through its binding energy and key residues. The initial screening of target peptides can be accomplished via activity prediction and molecular docking [106]. For example, umami peptides identified by Huang et al. formed stable complexes with the umami receptor T1R1/T1R3, confirming their umami perception intensity [107]. It is known that the peptide segment LSPSW has a good inhibitory effect on ACE [108].

## 4. Current Challenges and Future Prospects

Developing bioactive peptides from tuna faces challenges akin to those of other biologically derived peptides. Research on tuna-derived peptides is less extensive than that on peptides from other aquatic sources, with current studies primarily exploring antioxidant and antihypertensive properties. Tuna muscle and by-products are rich in diverse components; however, extracting active peptides requires precise separation techniques, as conventional methods often degrade peptides or diminish their bioactivity. The high cost of tuna raw materials and the complexity of extraction and purification processes significantly hinder large-scale production. Additionally, as functional food products, tuna-derived peptides must undergo thorough evaluations of long-term safety, including allergenicity and toxicological assessments. Their stability is also frequently compromised during processing and storage due to sensitivity to environmental factors like temperature and pH, necessitating further research to improve stability. While the bioactivity of certain tuna-derived peptides has been empirically validated, their precise mechanisms remain poorly understood. Further molecular biology and clinical studies are crucial to elucidate these mechanisms and confirm their efficacy. Despite practical challenges, these peptides hold significant potential for future development, thanks to advances in science and technology. Breakthroughs in proteomic technologies, such as mass spectrometry and high-throughput sequencing, have accelerated the identification, quantification, and structural characterization of bioactive peptides. Mass spectrometry decodes peptide modifications and sequence features, while solid-phase synthesis provides a scalable method for producing synthetic peptides, reducing dependence on natural sources. Future research on tuna-derived bioactive peptides should aim to thoroughly investigate structure-function relationships, clarifying how specific sequences dictate biological activities. This would provide a molecular basis for tailored applications. Expanding their use in functional foods, pharmaceuticals, and eco-friendly preservatives could reveal their multifunctional potential in various sectors. Concurrently, advancing extraction and synthesis technologies is essential to enhance production efficiency and economic viability. Additionally, systematic exploration of their cellular interactions and molecular mechanisms is crucial for understanding their mode of action, facilitating evidence-based optimization of therapeutic efficacy and safety in biomedical and nutritional applications.

## 5. Conclusions

Tuna-derived peptides are bioactive compounds obtained through extraction or synthesis, demonstrating antimicrobial, antioxidant, antihypertensive, and antitumor activities. These multifunctional properties highlight their potential for research and applications in food safety, pharmaceuticals, and nutraceuticals. Advances in biotechnology and materials science are expected to significantly enhance the value and applications of these peptides in the near future.

## Figures and Tables

**Table 1:** Antioxidant peptides derived from tuna.

Source	Extraction Site	Active Substance Name	Mechanism of Action	Reference
Bigeye tuna	Skin and bones	TSCP, TBCP	Inhibition of signaling pathways	[28]
Yellowfin tuna	Skin	PH, PWG, EL, AH, IR, HL	Inhibition of MPO activity	[29]
Yellowfin tuna	Trimmings	ACGSDGK	Inhibition of MPO activity	[30]
Skipjack Tuna	Bones	GADIVA, GAEGFIF	Scavenging free radicals	[31]
Skipjack Tuna	Dark muscle	YENGGG, EGYPWN, YIVYPG, WGDAGGGYY	Scavenging free radicals	[32]
Bigeye tuna	Dark muscle	APTDM	Scavenging free radicals	[33]
Skipjack Tuna	Skin	STG-AH	Scavenging free radicals	[34]
Skipjack Tuna	Roes	AEM, QDHK, YEA, AEHNH, YVM	Scavenging free radicals	[35]

**Table 2:** ACE inhibitory peptides derived from tuna.

Source	Extraction Site	Active Substance Name	IC50	Reference
Atlantic tuna	Muscle	LTGCP, YPKP	64.3 μM, 139.6 μM	[47]
Skipjack Tuna	Roes	MLVFAV	3.07 μM	[48]
Skipjack Tuna	Dark muscle	FPPDVA	87.11 ± 1.02 μM	[49]
Bigeye tuna	Dark muscle	WPEAAELMMEVDP	21.6 µM	[50]
Skipjack Tuna	Dark muscle	MWN, MEKS, MKKS, LPRS	0.328 ± 0.035, 0.527 ± 0.030, 0.269 ± 0.006, 0.495 ± 0.024 mg/mL	[51]
skipjack tuna	Milts	ICY, LSFR, IYSP	0.48, 0.59, 0.76 mg/mL	[53]
Skipjack Tuna	Muscle	SP, VDRYF	0.06 ± 0.01, 0.28 ± 0.03 mg/mL	[54]

**Table 3:** Bioactive peptides derived from tuna.

Source	Extraction Site	Active Substance Name	Activity	Reference
Yellowfin tuna	Dark muscles	KPLSeCPK	α-glucosidase inhibition	[76]
Skipjack tuna	Flesh of fish	FQLSAER, GEVDDSIQE, YEAFVK, KSIDDVEE	Umami peptide	[77]
Bluefin tuna	Skin	GPSGGGYDV	DPP-IV inhibitory peptide	[78]
Bluefin tuna	Skeletal myosin	LADW, MEIDD, VAEQE, EEAEGT	Umami peptide	[79]
Yellowfin tuna	Trimmings	HIAEEADRK AEQAESDKK	Immunomodulatory peptide	[80]
Skipjack tuna	Muscles	ACECD	XODI peptide	[81]
Skipjack tuna	Skin	SJGAP	Antimicrobial peptide	[82]
Bluefin tuna	Skeletal myosin	EEAGGATAAQIEM	Antiviral peptide	[83]
Yellowfin tuna	Pancreas	LLDF	Ache inhibitory peptide	[84]
Skipjack tuna	Dark muscles	APP, PPP, DPLL, EAVP, EAIP	DPP-IV inhibitory peptide	[85]

## Data Availability

Data available on request from the corresponding authors.
